# Fine-mapping the MHC locus in juvenile idiopathic arthritis (JIA) reveals genetic heterogeneity corresponding to distinct adult inflammatory arthritic diseases

**DOI:** 10.1136/annrheumdis-2016-210025

**Published:** 2016-12-20

**Authors:** A Hinks, J Bowes, J Cobb, H C Ainsworth, M C Marion, M E Comeau, M Sudman, B Han, Juvenile Arthritis Consortium for Immunochip, M L Becker, J F Bohnsack, P I W de Bakker, J P Haas, M Hazen, D J Lovell, P A Nigrovic, E Nordal, M Punnaro, A M Rosenberg, M Rygg, S L Smith, C A Wise, V Videm, L R Wedderburn, A Yarwood, R S M Yeung, S Prahalad, C D Langefeld, S Raychaudhuri, S D Thompson, W Thomson

**Affiliations:** 1 Arthritis Research UK Centre for Genetics and Genomics, Manchester Academic Health Science Centre, University Of Manchester, Manchester, UK; 2 NIHR Manchester Musculoskeletal Biomedical Research Unit, Central Manchester University Hospitals NHS Foundation Trust, Manchester, UK; 3 Center for Public Health Genomics and Department of Biostatistical Sciences, Wake Forest University School of Medicine, Winston-Salem, North Carolina, USA; 4 Center for Autoimmune Genomics and Etiology, Cincinnati Children's Hospital Medical Center, Cincinnati, Ohio, USA; 5 Divisions of Genetics and Rheumatology, Brigham and Women's Hospital, Harvard Medical School, Boston, USA; 6 Department of Convergence Medicine, University of Ulsan College of Medicine & Asan Institute for Life Sciences, Asan Medical Center, Seoul, Republic of Korea; 7 Division of Rheumatology and Division of Clinical Pharmacology, Toxicology, and Therapeutic Innovation, Children's Mercy-Kansas City, Kansas City, Missouri, USA; 8 Division of Allergy, Immunology and Paediatric Rheumatology, University of Utah, Salt Lake City, Utah, USA; 9 Department of Medical Genetics, Center for Molecular Medicine, University Medical Center Utrecht, Utrecht, The Netherlands; 10 German Center for Pediatric and Adolescent Rheumatology, Garmisch-Partenkirchen, Germany; 11 Division of Immunology, Department of Rheumatology, Boston Children's Hospital, Boston, Massachusetts, USA; 12 Division of Rheumatology, Cincinnati Children's Hospital Medical Center, Cincinnati, Ohio, USA; 13 Division of Rheumatology, Immunology and Allergy, Department of Medicine, Brigham and Women's Hospital and Harvard Medical School, Boston, USA; 14 Department of Paediatrics, University Hospital of North Norway, and UIT The Arctic University of Norway, Tromsø, Norway; 15 Arthritis Clinic Texas Scottish Rite Hospital for Children, Dallas, Texas, USA; 16 Department of Paediatrics, UT Southwestern Medical Center, Dallas, Texas, USA; 17 Division of Rheumatology, Department of Paediatrics, University of Saskatchewan, Saskatoon, Canada; 18 Department of Laboratory Medicine, Children's and Women's Health, NTNU - Norwegian University of Science and Technology, and St. Olavs University Hospital, Trondheim, Norway; 19 Sarah M. and Charles E. Seay Center for Musculoskeletal Research, Texas Scottish Rite Hospital for Children, Dallas, Texas, USA; 20 Department of Orthopaedic Surgery, Paediatrics, and McDermott Center for Human Growth and Development, UT Southwestern Medical Center, Dallas, Texas, USA; 21 Arthritis Research UK Centre for Adolescent Rheumatology, UCL GOS Institute of Child Health, University College London, London, UK; 22 NIHR-Great Ormond Street Hospital Biomedical Research Centre, London, UK; 23 The Hospital for Sick Children and University of Toronto, Toronto, Canada; 24 Department of Paediatrics, Emory University School of Medicine, and Children's Healthcare of Atlanta, Atlanta, USA; 25 Program in Medical and Population Genetics, Broad Institute of MIT and Harvard, Cambridge, USA; 26 Department of Medicine, Karolinska Institutet and Karolinska University Hospital Solna, Stockholm, Sweden

**Keywords:** Juvenile Idiopathic Arthritis, Autoimmune Diseases, Rheumatoid Arthritis, Gene Polymorphism

## Abstract

**Objectives:**

Juvenile idiopathic arthritis (JIA) is a heterogeneous group of diseases, comprising seven categories. Genetic data could potentially be used to help redefine JIA categories and improve the current classification system. The human leucocyte antigen (HLA) region is strongly associated with JIA. Fine-mapping of the region was performed to look for similarities and differences in HLA associations between the JIA categories and define correspondences with adult inflammatory arthritides.

**Methods:**

Dense genotype data from the HLA region, from the Immunochip array for 5043 JIA cases and 14 390 controls, were used to impute single-nucleotide polymorphisms, HLA classical alleles and amino acids. Bivariate analysis was performed to investigate genetic correlation between the JIA categories. Conditional analysis was used to identify additional effects within the region. Comparison of the findings with those in adult inflammatory arthritic diseases was performed.

**Results:**

We identified category-specific associations and have demonstrated for the first time that rheumatoid factor (RF)-negative polyarticular JIA and oligoarticular JIA are genetically similar in their HLA associations. We also observe that each JIA category potentially has an adult counterpart. The RF-positive polyarthritis association at HLA-DRB1 amino acid at position 13 mirrors the association in adult seropositive rheumatoid arthritis (RA). Interestingly, the combined oligoarthritis and RF-negative polyarthritis dataset shares the same association with adult seronegative RA.

**Conclusions:**

The findings suggest the value of using genetic data in helping to classify the categories of this heterogeneous disease. Mapping JIA categories to adult counterparts could enable shared knowledge of disease pathogenesis and aetiology and facilitate transition from paediatric to adult services.

## Introduction

Juvenile idiopathic arthritis (JIA), the most common arthritic disease of childhood, is a heterogeneous group of diseases. The current International League of Associations for Rheumatology (ILAR) classification system defines seven categories based on clinical features, including an undifferentiated category for cases that do not fall into one of the defined categories.^1^ Genetic data could be used to help define JIA categories and improve the current classification system. Prior studies of the best established genetic risk factor for JIA, the major histocompatibility region (MHC) on chromosome 6, have been in modest sample sizes.^2^ ^3^ The development of methods for imputation of classical human leucocyte antigen (HLA) alleles and amino acids^4^ from genotyping array data enables a comprehensive and cost-effective approach for generating HLA typing on much larger JIA cohorts. We sought to use this powerful approach to dissect and refine the HLA associations of the heterogeneous JIA categories.

While there are considerable clinical similarities between some JIA categories and adult inflammatory arthritides, there is also substantial heterogeneity. Hence, we sought to compare the associations across the MHC region observed in JIA cohorts with those observed in adult inflammatory arthritides, such as rheumatoid arthritis (RA).^5^ ^6^ Furthermore, some categories of JIA have obvious adult counterparts (eg, enthesitis-related arthritis (ERA) and adult ankylosing spondylitis (AS), or juvenile psoriatic arthritis (jPsA) with psoriatic arthritis), the most common categories of JIA, oligoarthritis and rheumatoid factor (RF)-negative polyarthritis, do not appear to map to any adult form of disease. Mapping each of the JIA categories to RA and other adult inflammatory arthritic diseases could have many benefits including enhanced understanding of the genetic basis and etiopathogenesis of inflammatory arthritis in general, allow extrapolation of results from clinical trials in adult inflammatory arthritis to paediatric counterparts to improve the therapy of JIA, and facilitate smooth transition of paediatric patients to adult care providers with consistent clinical designations.

The goals of this study were threefold, to use comprehensive MHC fine-mapping genetic data to refine HLA associations across each JIA category, to assess correspondences between the JIA categories and finally compare associations with adult inflammatory arthritic diseases.

## Methods

### Subjects

All cohorts comprised individuals from populations of European descent from the USA, UK, Canada, Norway and Germany. Descriptions of the datasets can be found in the online supplementary information. The total dataset prequality control comprised all JIA categories and included 5737 patients with JIA and 16 403 controls genotyped for 191 494 markers.

10.1136/annrheumdis-2016-210025.supp1 supplementary data

### Genotyping and quality controls

Samples were genotyped using ImmunoChip, a custom-made Illumina Infinium array, described previously.^7^ The ImmunoChip includes dense coverage of the HLA region and 186 additional non-HLA loci. Genotyping was performed according to Illumina's protocols at labs in Hinxton, UK, Manchester, UK, Cincinnati, USA, Utah, USA, Charlottesville, USA, New York, USA, Brisbane, Australia and Toronto, Canada. The Illumina GenomeStudio GenTrain V.2.0 algorithm was used to recluster all 22 140 samples together.

Single-nucleotide polymorphisms (SNPs) were initially excluded if they had a call rate <98% and a cluster separation score of <0.4. A SNP was subsequently removed from the primary analysis, if it exhibited significant differential missingness between cases and controls (p<0.05), had significant departure from Hardy-Weinberg equilibrium (p<0.000001 in cases or p<0.01 in controls), or had a minor allele frequency (MAF) <0.01. Based on the SNPs that passed the above quality control thresholds, samples were then excluded for call rate <98%, or if there were inconsistencies between recorded and genotype-inferred gender or excess heterozygosity on the autosomes. Duplicates and first-degree or second-degree relatives were removed based on identity-by-descent statistics computed using the programme KING.^8^ Admixture estimates were computed on the remaining samples while including the HapMap phase III individuals (CEU, YRI and CHB) as reference populations using the software ADMIXTURE.^9^ The admixture estimates were then used to identify and remove genetic outliers. Three of these admixture estimates were included as covariates in the logistic regression (association) analysis to account for within-sample variation.

### HLA imputation

The markers spanning 29–34 Mb (hg build19) on chromosome 6 which encompasses the HLA region were extracted from the post-QC Immunochip dataset. Cases and controls were imputed together using SNP2HLA (V.1.0) (http://www.broadinstitute.org/mpg/snp2hla/).^4^ This is a robust approach which enables imputation of classical HLA alleles as well as specific amino acid positions within HLA alleles, which may play an important functional role. The method uses a large reference dataset collected by the type 1 diabetes genetics consortium^10^ (n=5225). This dataset has gold-standard HLA typing and high SNP density, thus using linkage disequilibrium patterns around SNPs and classical HLA alleles enables the inference of classical HLA alleles, amino acids and SNPs across the region based on the SNP data generated from Immunochip, an approach successfully used by a number of researchers.^5^ ^6^ ^11^ ^12^ Post-imputation QC included removing variants with a MAF <0.01 and variants with an r^2^ <0.8. The dosage output, which accounts for imputation uncertainty, was used for the association analyses.

To assess the quality of the imputation, a proportion of the UK and the US JIA cases have two-digit and four-digit *HLA-DRB1* typing available (n=1562) performed using a semi-automated, reverse dot-blot method,^2^ ^3^ which was used to calculate the proportion of accurately imputed two-digit and four-digit *HLA-DRB1* alleles. In addition, the *DRB1* two-digit and four-digit allele frequencies were compared between genotyped and imputed HLA allele calls.

### Association analysis of HLA alleles and amino acid polymorphisms

To compare the differences and similarities of HLA associations across the different JIA categories, genetic correlation of the MHC region between the categories was calculated using bivariate analysis^13^ implemented using GCTA.^14^ This analysis first calculates the genetic variance (heritability) of each category and then calculates the genetic correlation between the categories across the HLA region. High correlation suggests similarities or pleiotropy between the two categories compared. This analysis requires independent controls for the two categories being compared and therefore the controls were randomly assigned to the two categories, splitting equally, taking into account the proportions of controls from each population.

HLA variants were binary coded (presence or absence) and included SNPs and two-digit and four-digit HLA alleles. Association analysis was performed using logistic regression in R, using dosage data (genotype probabilities), which takes into account imputation uncertainty. For the analysis of each JIA category, the total control dataset was used for each comparison. HLA amino acid polymorphisms have multiple residues at each position and were analysed using the omnibus test. This is a log-likelihood ratio test comparing the likelihood of the null model against the likelihood of the fitted model, which gave a p value assessing the improvement in fit of the model, the deviance is calculated (−2×the log likelihood ratio), which follows a χ^2^ distribution with m−1 degrees of freedom (where m is the number of HLA variant alleles).^5^ Three of the admixture estimates were included as covariates to account for potential population stratification.

To look for independent effects across the HLA region, conditional analysis was performed. Logistic regression, as described above, was performed to identify the most associated marker. Then this marker was used as a covariate in the model and logistic regression repeated. This analysis was continued sequentially in a forward stepwise approach until no variant satisfied the genome-wide significance threshold (conditioned p<5×10^−8^). When the covariate was an amino acid, all multi-allelic variants of the amino acid were included as covariates, excluding the most frequent variant. To look for additional effects outside *HLA-DRB1*, we included all two-digit and four-digit HLA-DRB1 alleles within the model and looked for any residual effects.

To confirm the results of the conditional analysis and to check that there were no other combinations of variants that better fitted the models derived from the forward stepwise approach, described above, we exhaustively tested all possible combinations of 2, 3 and 4 amino acid positions, including the three admixture estimates as covariates. For each combination we calculated deviation from the null hypothesis, which included only the admixture covariates. To assess the improvement in the model fit we also calculated the improvement in the Akaike information criterion (ΔAIC), and also the improvement in the Bayesian information criterion (ΔBIC).

We used a disease prevalence of 0.001 to estimate the variance explained (h^2^) by the HLA region and some of the independent effects and compared with the estimate calculated for all Immunochip, implemented using GCTA.^14^

## Results

### HLA imputation

Post-QC data was available for 6920 SNPs, 335 amino acids and 171 HLA alleles in 5043 JIA cases and 14 390 healthy controls (see online supplementary table S1). A detailed breakdown of the JIA cases by ILAR category is shown in table 1, and by population and gender in online supplementary table S2.

**Table 1:** Breakdown of the total JIA cohort by ILAR category

ILAR category	Number
All JIA	5043
Systemic JIA	373
Oligoarthritis	
Persistent	1751
Extended	658
RF-negative polyarthritis	1525
RF-positive polyarthritis	337
Enthesitis-related arthritis	183
Juvenile psoriatic arthritis	112
Undifferentiated	104
Combined oligoarthritis and RF-negative polyarthritis dataset	3934

ILAR, International League of Associations for Rheumatology; JIA, juvenile idiopathic arthritis; RF, rheumatoid factor.

For a proportion of the UK and US JIA cases (n=1562), two-digit and four-digit HLA-DRB1 classical typing was available. The accuracy of the imputed data was calculated as 97.9% for two-digit and 89.3% for four-digit alleles, which is similar to those calculated in previous studies in RA.^5^ A detailed analysis strategy is shown in online supplementary figure S1.

### Bivariate analysis to look for genetic correlation between the JIA categories

**Figure 1:**
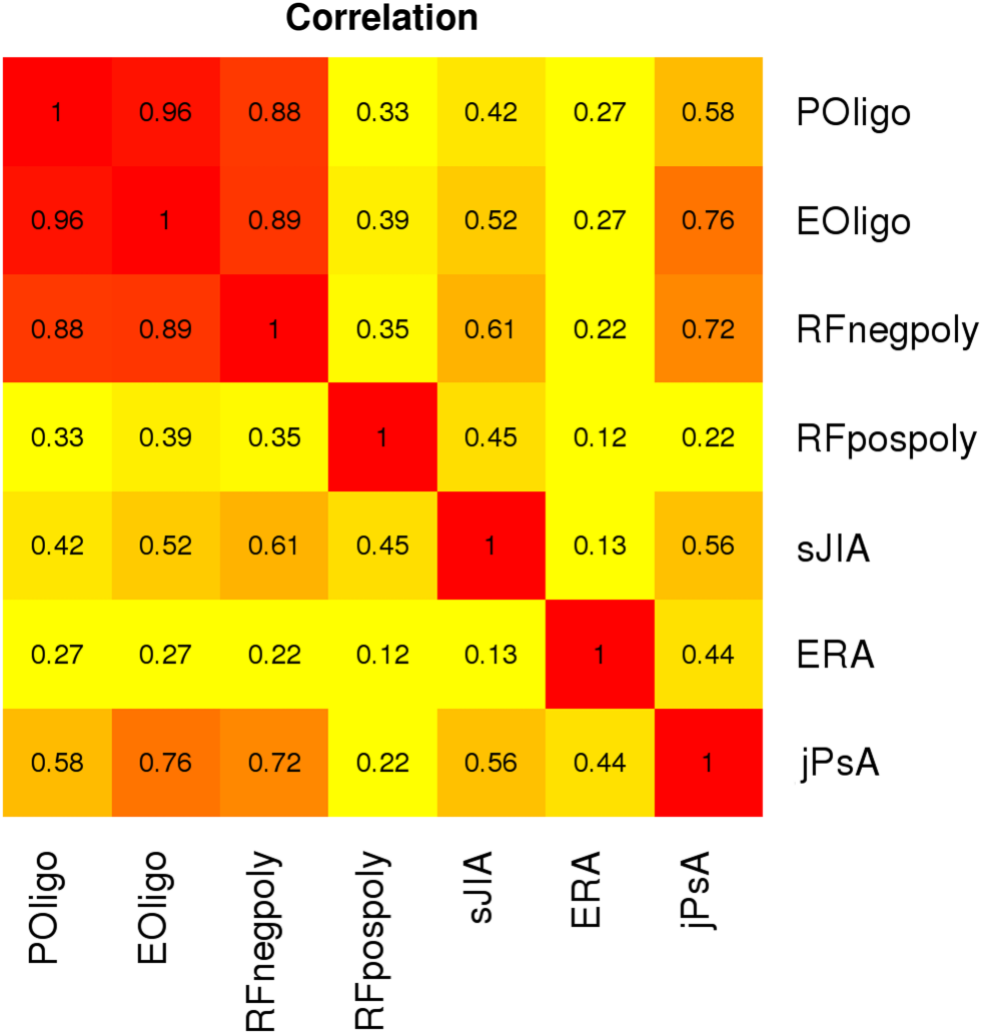
Heatmap showing genetic correlation for human leucocyte antigen (HLA) between the juvenile idiopathic arthritis (JIA) categories. Each square shows the level of correlation between each JIA category. With a scale of red colour representing higher correlation through orange to yellow for low correlation. The numbers within the squares represent the correlation values ranging from 0 to 1 for high correlation. Note that this plot is symmetrical. POligo, persistent oligoarthritis; EOligo, extended oligoarthritis; RF, rheumatoid factor; RFnegpoly, RF-negative polyarthritis; RFpospoly, RF-positive polyarthritis; sJIA, systemic JIA; ERA, enthesitis-related arthritis; jPsA, juvenile psoriatic arthritis.

We performed bivariate analysis to calculate the estimated HLA region genetic correlation between each pair of JIA categories (figure 1). The heritability for each category estimated from the bivariate analysis was similar to that estimated from univariate analyses performed in the total dataset (see online supplementary tables S3 and S4). The estimates of correlation between each pair of JIA categories showed a surprisingly strong correlation between the most common categories of JIA: RF-negative polyarthritis, persistent and extended oligoarthritis (rG>0.88). In contrast, the correlations between these three categories and the other categories of JIA were lower (figure 1).

### Association analysis of HLA markers

**Figure 2:**
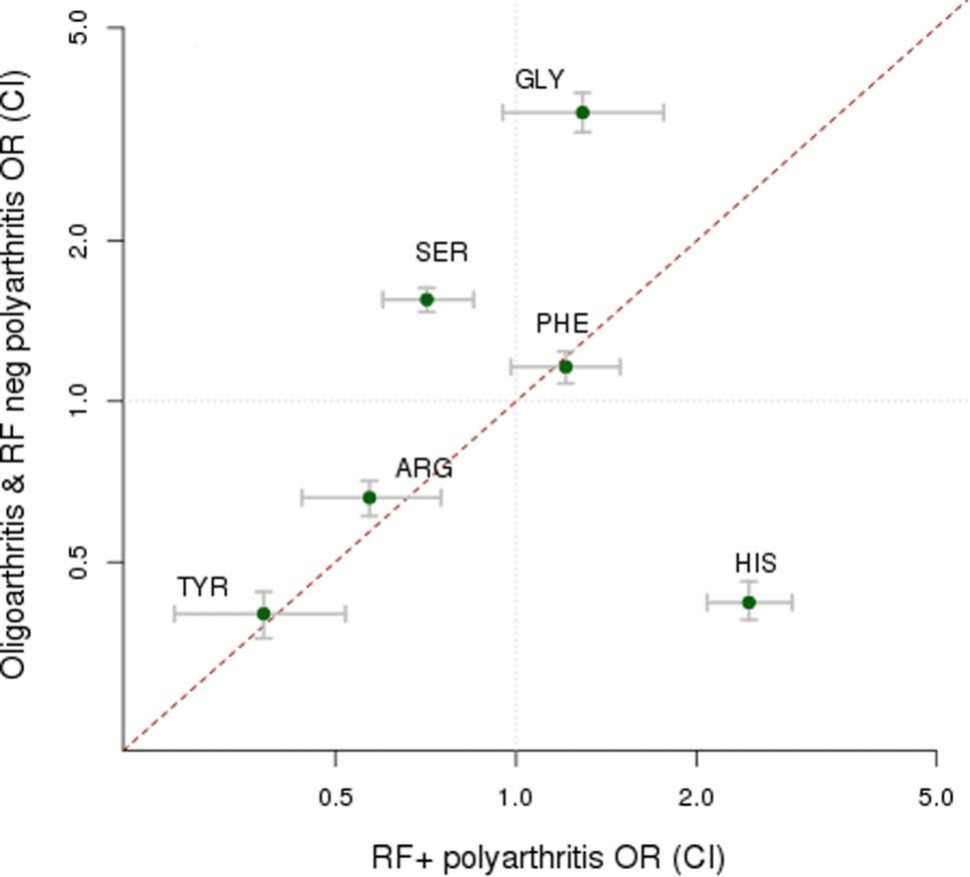
Different effect sizes (ORs and 95% CIs) for amino acid residues at human leucocyte antigen (*HLA*)*-DRB1* position 13 between the combined dataset of oligoarthritis and rheumatoid factor (RF)-negative polyarthritis compared with those for RF-positive polyarthritis.

After conducting primary association analysis of all 7426 variants, in each of the seven JIA categories (table 2), we observed that for oligoarthritis and both RF-positive and RF-negative polyarthritis the strongest association was with *HLA-DRB1* amino acid position 13. However, for oligoarthritis and RF-negative polyarthritis, the most common categories of JIA, glycine13 confers the strongest risk; serine13 also confers a risk effect but histidine13 is protective. By contrast, in RF-positive polyarthritis, it is histidine13 that confers the strongest risk and serine13 confers a strong protective effect (see figure 2, online supplementary table S5 and supplementary figure S2). When the effect estimates for the histidine13 residue in the associated JIA categories were compared using multinomial logistic regression, strong protective effects were observed in persistent and extended oligoarthritis, with no significant difference in the effect estimates (p=0.63). There was a slightly weaker, protective effect for RF-negative polyarthritis compared with that for persistent and extended oligoarthritis (p<0.05). Importantly, there was a significantly different risk effect in RF-positive polyarthritis compared with RF-negative polyarthritis, persistent and extended oligoarthritis. The remaining JIA categories had distinct HLA associations from these common categories. The most significant association in systemic JIA (sJIA) was for HLA-DRB1*11 and for the ERA category was HLA-B*27. For jPsA, no associations reached genome-wide level of significance (p<5×10^−8^).

**Table 2:** Primary association of the HLA region in JIA ILAR categories

Category	HLA variant primary association	Position	p Value	Amino acid residue	OR	95% CI
Systemic JIA	HLA-DRB1*11	32660042	3.41×10^−11^		2.09	1.67 to 2.59
Persistent oligoarthritis	*HLA-DRB1* AA pos 13	32660109	9×10^−256^	Glycine	2.72	2.41 to 3.08
Extended oligoarthritis	*HLA-DRB1* AA pos 13	32660109	1.05×10^−104^	Glycine	2.87	2.4 to 3.42
RF-negative polyarthritis	*HLA-DRB1* AA pos 13	32660109	4.29×10^−99^	Glycine	2.02	1.76 to 2.32
RF-positive polyarthritis	*HLA-DRB1* AA pos 13	32660109	3.65×10^−31^	Histidine	2.44	2.0 to 2.97
ERA	HLA-B*27	31431272	8.81×10^−98^		1.71	1.32 to 2.24

ERA, enthesitis-related arthritis; HLA, human leucocyte antigen; ILAR, International League of Associations for Rheumatology; JIA, Juvenile idiopathic arthritis; RF, rheumatoid factor.

### Investigation of multiple effects within the region

**Figure 3:**
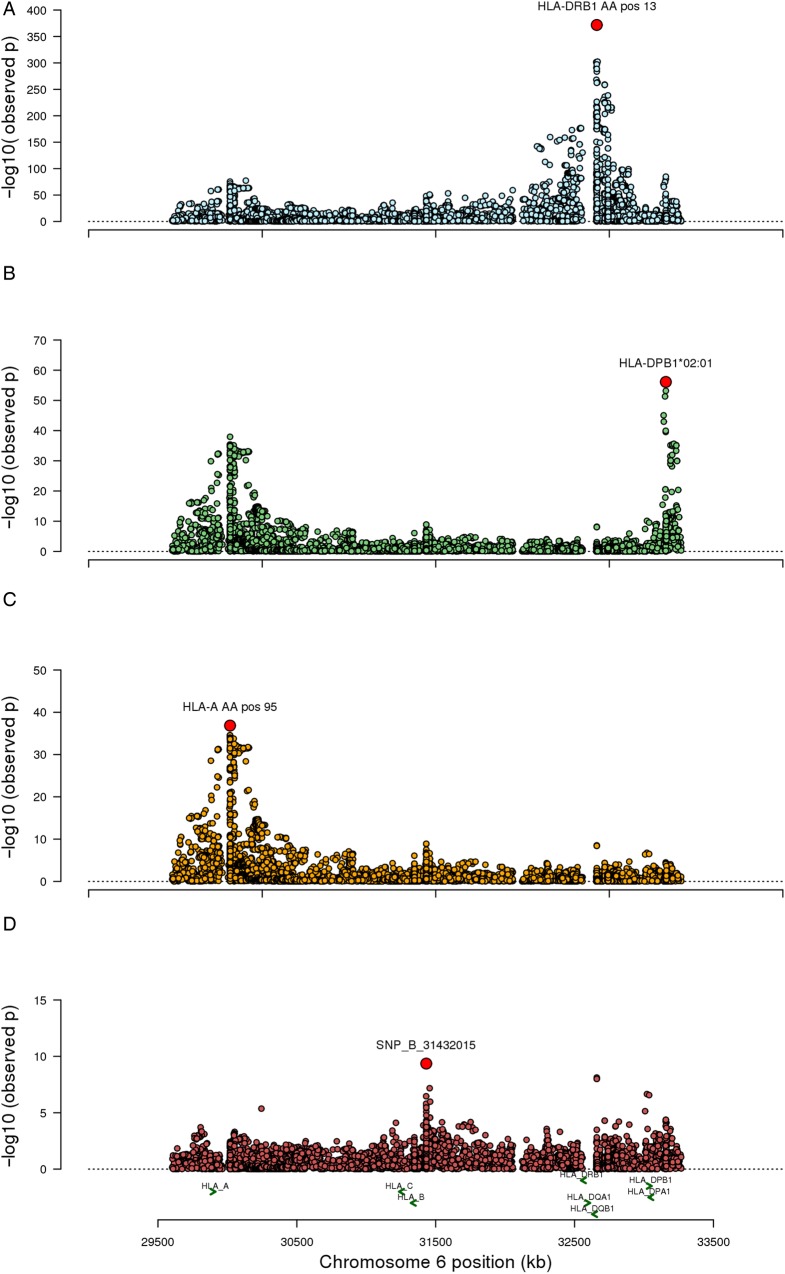
Analysis in the combined dataset of persistent and extended oligoarthritis and rheumatoid factor (RF)-negative polyarthritis, evidence for multiple independent effects across the major histocompatibility region (MHC). (A) Association results for all human leucocyte antigen (HLA) markers, *HLA-DRB1* amino acid position 13 showed the strongest association (p<10^−377^). (B) Conditioning on all *HLA-DRB1* two-digit and four-digit alleles, HLA-DPB1*02:01 was associated (p<10^−57^). (C) Conditioning on all *HLA-DRB1* two-digit and four-digit alleles and HLA-DPB1*02:01, *HLA-A* amino acid position 95 was associated (p<10^−37^). (D) Conditioning on all *HLA-DRB1* two-digit and four-digit alleles, HLA-DPB1*02:01 and *HLA-A* amino acid position 95, *HLA-B* amino acid position 152 was associated (p<10^−10^).

Observing that oligoarthritis and RF-negative polyarthritis showed similar HLA associations and evidence for pleiotropy from the bivariate analysis in GCTA,^13^ ^14^ these categories were combined to increase power for further analyses (total sample size=3934). To look for independent genetic effects across the HLA region, we conditioned on the most associated marker, *HLA-DRB1* amino acid 13 and detected a second independent effect within *HLA-DRB1* at amino acid position 67 (omnibus p=7.01×10^−83^). Further conditioning revealed separate effects at amino acid positions 181 (omnibus p=3.33×10^−22^) and 71 (omnibus p=1.16×10^−8^) (see online supplementary table S6 and supplementary figure S3). Conditioning on all two-digit and four-digit *HLA-DRB1* alleles found independent effects at HLA-DPB1*02:01, *HLA-A* amino acid 95 and *HLA-B* amino acid 152 (see online supplementary table S6 and figure 3). All possible combinations of 2, 3 and 4 amino acids in *HLA-DRB1* were exhaustively tested. *HLA-DRB1* amino acids 13 and 67 were the most strongly associated of all possible 2 amino acid combinations. However, for both the 3- amino acid and 4-amino acid combinations, there were two other combinations that had a better model, according to deviance from the null hypothesis, compared with the combination of *HLA-DRB1* amino acids at positions 13, 67 and 181 or for *HLA-DRB1* at positions 13, 67, 181 and 71, the 3- and 4-amino acid models that might have been expected to be the most significant considering the results of the conditional analysis. Therefore, while there appears to be multiple independent effects within the *HLA-DRB1* gene, we can only be confident in the *HLA-DRB1* amino acids at positions 13 and 67.

### Variance explained by the HLA region

We calculated the proportion of variance explained by the independent HLA variants in the combined oligoarthritis and RF-negative polyarthritis dataset (see table 3 and online supplementary table S4) and found that the total HLA region explained 8% of the total phenotypic variance, with the *HLA-DRB1* region, driven by the amino acid at position 13, contributing 50% of variance explained by the HLA region.

**Table 3:** Heritability estimates for HLA and various alleles and All Immunochip in the combined oligoarthritis and RF-negative polyarthritis dataset (n=3934)

Cohort	h^2^_SNP_(SE)
All Immunochip	0.17 (0.006)
Total HLA region	0.08 (0.007)
All Immunochip without HLA	0.09 (0.006)
*HLA-DRB1* 2D/4D alleles	0.04 (0.008)
*HLA-DPB1* 2D/4D alleles	0.02 (0.005)
*HLA-A* 2D/4D alleles	0.006 (0.002)
*HLA-DRB1* amino acid position 13	0.04 (0.02)
*HLA-DRB1* amino acid positions 13 and 67	0.04 (0.02)

h^2^_SNP_, heritability; HLA, human leucocyte antigen; RF, rheumatoid factor.

### Comparison with adult inflammatory arthritic diseases

We compared our HLA association findings across JIA categories with those of adult inflammatory arthritic diseases (see online supplementary table S7). In seropositive RA, Raychaudhuri *et al* showed multiple independent associations within the *HLA-DRB1* gene at three amino acid positions (11, 71 and 74) and also independent associations at amino acid position 9 in *HLA-B* and amino acid position 9 in *HLA-DPB1*.^5^ The DRB1 amino acid at position 11 is in strong linkage disequilibrium with the amino acid at position 13, which makes it difficult to assign causality to one or the other. In this study, oligoarthritis and polyarthritis each showed association with *HLA-DRB1* amino acid at position 13. If the ORs of the residues at *HLA-DRB1* amino acid position 13 for paediatric and adult arthritic diseases are compared, the combined oligoarthritis and RF-negative polyarthritis dataset shows similar ORs to that seen in seronegative RA^6^ (see online supplementary figure S4), suggesting that these JIA categories could potentially have an adult counterpart. Likewise, in RF-positive polyarthritis, the histidine residue at position 13 at *HLA-DRB1* confers the greatest risk for disease and, unsurprisingly, this mirrors the association in seropositive RA^5^ (see online supplementary figure S4). For the ERA category, as expected the most significant association was for HLA-B*27, the same HLA allele found in AS.

10.1136/annrheumdis-2016-210025.supp2 Supplementary table 7

10.1136/annrheumdis-2016-210025.supp3 Supplementary table 8

## Discussion

This is the largest investigation of association of the HLA region with JIA and its categories to date, exploiting novel imputation strategies we have observed differences and similarities between HLA associations for the different categories. The most common and also the most clinically homogeneous categories of JIA, oligoarthritis and RF-negative polyarthritis, showed strong genetic correlation across the HLA region supporting our previous approaches of combining these categories for genetic studies.^15^ Combined analysis of these categories show they share association across the HLA region with strong association for *HLA-DRB1* amino acid position 13. The results for these combined categories are consistent with previous findings investigating association of classical HLA alleles in JIA. For example, there is previous evidence for association of HLA-DRB1*08 and the HLA alleles that lie on this haplotype, with oligoarthritis and RF-negative polyarthritis.^2^ ^3^ At *HLA-DRB1* amino acid position 13, the glycine residue lies on the HLA-DRB1*08 haplotype. The association with the amino acids is much stronger than that for the classical HLA allele (see online supplementary figure S3A). These combined categories also show multiple independent effects across the region, at *HLA-DRB1* amino acid position 67, additional association at HLA-DPB1*02:01, an effect at *HLA-A* and at *HLA-B*. There is previous evidence for association of HLA-DPB1*0201 and HLA-A*02 (we found stronger association considering amino acids, specifically a valine residue at *HLA-A* amino acid position 95 in HLA-A*02). Previous studies have failed to demonstrate an association with *HLA-B*, which are apparent only with the additional samples available for this study.

A striking finding has been the shared association of *HLA-DRB1* amino acid position 13 for both paediatric and adult diseases. It is known that amino acid position 13 is involved in shaping the peptide-binding pocket 4 of HLA-DRB1.^16^ We find that the association in the combined oligoarthritis and RF-negative polyarthritis dataset mirrors the findings seen in seronegative RA and similarly, in RF-positive polyarthritis, the findings correspond to the association in seropositive RA. Interestingly, the magnitudes of associations are stronger in the paediatric diseases compared with adult, suggesting the paediatric disease is more genetically driven.

We then further compared the associations seen in each of the other JIA categories with those of their proposed adult counterparts. Based on clinical features, it is likely that sJIA would map to adult Still's disease, but there is currently no HLA genetic data to support or refute this. The most significant association in sJIA was for HLA-DRB1*11, consistent with recent findings from a large genome-wide association study for sJIA, which used an overlapping set of samples.^17^ Previous studies of a HLA association with sJIA had yielded conflicting results, but there is now clear evidence for association of the HLA region with this category of JIA. Data from the current study also show that the association is distinct to that seen in the other categories. This supports emerging evidence that sJIA is a distinct disease, with less of an autoimmune phenotype and displaying auto-inflammatory features^18^ and builds on previous genetic evidence, which reported no association with another well-established JIA susceptibility gene, *PTPN22*, in sJIA.^19^ Unsurprisingly, the strongest association seen in ERA, HLA-B*27, is the same as adult AS.^20^ Although no associations reaching genome-wide level of significance (p<5×10^−8^) were seen in jPsA, the most significant HLA alleles were HLA-DQA1*0401 (p=0.0001), HLA-DRB1*08 (p=0.0003) and HLA-DQB1*0402 (p=0.0008), which all lie on the same haplotype. The established HLA association in adult-onset PsA is HLA-C*0602,^21^ which is also the primary HLA association in psoriasis,^22^ was also modestly associated in this study (p=0.008). There was also evidence in jPsA for association with HLA-B*27 (p=0.003), the HLA allele that is the most significant in ERA. The mixed HLA associations in jPsA may suggest some misclassification such that the jPsA samples may contain some individuals from oligoarthritis, RF-negative polyarthritis or ERA categories. This is perhaps not surprising given that jPsA is difficult to classify, and that some of the jPsA classification criteria have been disputed.^23^

The results of this study have important implications for understanding disease pathogenesis, aetiology and potential future therapeutic strategies for JIA categories. Despite the development of a classification system, heterogeneity still exists within the ILAR categories. This heterogeneity of JIA remains a key challenge to paediatric rheumatologists; however, these results may inform the debate on classification and help define a more biological-driven and molecular-driven classification system. We show clear differences among many of the categories in terms of their HLA associations, but here we have also shown that the most common categories of JIA, oligoarthritis and RF-negative polyarthritis, are genetically similar and also notably similar to adult-onset seronegative RA. It is only relatively recently that the heterogeneous nature of adult RA has been recognised, with seronegative RA less common than seropositive RA.^24^ ^25^ There are no specific therapeutic strategies for seronegative RA at this time, but given the rarity of this subphenotype of RA and the JIA categories individually, this study suggests that further comparisons of genetic studies for these diseases could help identify novel pathways and targets for therapy for both adult-onset and childhood-onset forms of inflammatory arthritis.
