# Supplementary material for: Fine-mapping the MHC locus in juvenile idiopathic arthritis (JIA) reveals genetic heterogeneity corresponding to distinct adult inflammatory arthritic diseases
Source: Ann Rheum Dis. 2016 Dec 20;76(4):765–72. doi: 10.1136/annrheumdis-2016-210025 (PMC5530326; doi:10.1136/annrheumdis-2016-210025)

Supplementary information

Juvenile Arthritis Consortium for Immunochip (JACI): Hannah C. Ainsworth, Robert Andrews, Mara L. Becker, John F. Bohnsack, John Bowes, Matthew A. Brown, Milton Brown, Wei-Min Chen, Joanna Cobb, Patrick Concannon, Mary E. Comeau, Panos Deloukas, Sarah Edkins, Stephen Eyre, Patrick M. Gaffney, Stephen L. Guthery, Joel M. Guthridge, Johannes Peter Haas, Melissa Hazen, Anne Hinks, Sarah E. Hunt, Judith A. James, Carl D. Langefeld, Daniel J. Lovell, Miranda C. Marion, Kathy L. Moser, Peter A. Nigrovic, Ellen Nordal, Suna Onengut-Gumuscu, Sampath Prahalad, Marilyn Punaro, Carlos D. Rosé, Alan M. Rosenberg, Stephen S. Rich, Marite Rygg, Satria Sajuthi, Kathryn JA. Steel, Marc Sudman, Wendy Thomson, Susan D. Thompson, Vibeke Videm, Edward K. Wakeland, Carol A. Wallace, Lucy R. Wedderburn, Carol Wise, Patricia Woo, Rae SM. Yeung.

Biologically Based Outcome Predictors in JIA (BBOP)[#], Boston Children's JIA Registry[#], British Society of Paediatric and Adolescent Rheumatology (BSPAR) study group[#], Childhood arthritis prospective study (CAPS)[#], Childhood Arthritis Response to Medication Study (CHARMS)[#], German society for Pediatric Rheumatology (GKJR)[#], JIA gene expression studies[#], NIAMS JIA genetic registry[#], Nordic JIA study[#], TREAT study[#], Understanding TNF Therapy in JIA Project[#], United Kingdom Juvenile Idiopathic Arthritis Genetics Consortium (UKJIAGC)[#]

[#]Consortia

Biologically Based Outcome Predictors in JIA (BBOP): Adam Baxter-Jones, Susanne Benseler, Sasha Bernatsky, David Cabral, Bonnie Cameron, Sarah Campillo, Gaëlle Chédeville, Anne-Laure Chetaille, Ang Cui, Paul Dancey, Joan Dietz, Andrea Doria, Ciaran Duffy, Karen Duffy, Janet Ellsworth, Simon Eng, Brian Feldman, Sarah Finch, Marvin Fritzler, Anna Goldenberg, John R Gordon, Jaime Guzman, David Hart, Daniel Hogan, Kristen Houghton, Adam Huber, Nicole Johnson, William Kulyk, Anthony Kusalik, Ron M Laxer, Loren Matheson, Deborah Levy, Bindu Nair, Kiem G Oen, Punam Pahwa, Ross E Petty, Suzanne Ramsey, Martin Reed, Elham Rezaei, Alan M Rosenberg, Johannes Roth, Dax Rumsey, Stephen W Scherer, Heinrike Schmelling, Rayfel Schneider, Jennifer Stinson, Rosie Scuccimarri, Claire St-Cyr, Elizabeth Stringer, Brett Trost, Shirley ML Tse, Lori B Tucker, Hassan Vatanparast, Sheldon Wiebe, Richard F. Wintle, Rae SM Yeung.

Boston Children's JIA Registry: Peter A. Nigrovic, Fatma Dedeoglu, Robert C. Fuhlbrigge, Melissa M. Hazen, Lauren A. Henderson, Erin Janssen, Susan Kim, Mindy S. Lo, Mary Beth F. Son, Robert P. Sundel, Irit Tirosh, Heather O. Tory.

British Society of Paediatric and Adolescent Rheumatology (BSPAR) study group: Mario Abinum, A. Bell, Alan W. Craft, Esther Crawley, Joel David, Helen Foster, Janet Gardener-Medwin, Jane Griffin, Ann Hall, M. Hall, Ariane L. Herrick, Peter Hollingworth, Lennox Holt, Stan Jones, Gillian Pountain, Clive Ryder, Tauny Southwood, I. Stewart, Helen Venning, Lucy R. Wedderburn, Patricia Woo, Sue Wyatt.

Childhood Arthritis Prospective Study (CAPS): Eileen Baildam, Nick Bishop, Lynsey Brown, Joanne Buckley, Alice Chieng, Roberto Carrasco, Joanna Cobb, Lucy Cook, Joyce Davidson, Annette Duggan, Michael Eltringham, Helen Foster, Elizabeth Friel, Mark Friswell, Janet Gardner-Medwin, Paul Gilbert, Vikki Gould, Kelly Hadfield, Kimme Hyrich, Julie Jones, Sham Lal, Mark Lay, Gabrielle Lloyd, Olivia Lloyd, Carol Lydon, Natasha Makengo, Ann McGovern, Alexandra Meijer, Nicola Mills-Wierda,

Theresa Moorcroft, Vicki Price, Liang Qiao, Kay Riding, Jane Sim, Tauny Southwood, Wendy Thomson, Maureen Todd, Susan Tremble, Katharine Venter, Debbie Wade, Peter Ward, Sharon Watson, Gwen Webster, Lucy R Wedderburn, Jadranka Zelenovic.

Childhood Arthritis Response to Medication Study (CHARMS): Katrin Burkle, Joanna Cobb, Angela Etheridge, Paul Gilbert, Anne Hinks, Shashi Hirani, Laura Kassoumeri, Sham Lal, Laura Melville, Halima Moncrieffe, Kathleen Mulligan, Stanton Newman, Fiona Patrick, Tauny Southwood, Wendy Thomson, Simona Ursu, Lucy R Wedderburn, Pamela Whitworth, Patricia Woo.

German Society for Pediatric Rheumatology (GKJR): Guenther Dannecker, Gerd Ganser Johannes Peter Haas, Hartmut Michels.

JIA gene expression studies: Mara L. Becker, Robert A. Colbert, Jason Dare, Beth S. Gottleib, Thomas A. Griffin, Alexi Grom, Norman T. Ilowite, Daniel J. Lovell, Halima Moncrieffe, Peter A. Nigrovic, Judy Ann Olsen, Sampath Prahalad, Margalit Rosenkranz, David D. Sherry, Susan D. Thompson.

NIAMS JIA genetic registry: John F. Bohnsack, Gloria Higgins, Marissa Klein-Gittelman, David N. Glass, T. Brent Graham, Thomas A. Griffin, Paula W. Morris, Natasha Ruth, Murray H. Passo, Sampath Prahalad, Stephen J Spalding, Susan D. Thompson.

Nordic JIA study, Norwegian extended subcohort: Marite Rygg, Ellen Nordal.

The Nord-Trøndelag Health Study (HUNT): Vibeke Videm, Matthew A. Brown.

TREAT study: Carol A. Wallace, Edward H. Giannini, Stephen J. Spalding, Phillip J. Hashkes, Kathleen M. O'Neil, Andrew S. Zeft, Illona S. Szer, Sarah Ringold, Hermine I. Brunner, Laura E. Schanberg, Robert P. Sundel, Diana Milojevic, Marilynn G. Punaro, Peter Chira, Beth S. Gottlieb, Gloria C. Higgins, Norman T. Ilowite, Yukiko Kimura, Stephanie Hamilton, Anne Johnson, Bin Huang, Daniel J. Lovell.

Understanding TNF Therapy in JIA Project: Daniel J. Lovell, Anne L. Johnson, Steven J. Spalding, Andrew Zeft, Beth S. Gottlieb, Paula W. Morris, Yukiko Kimura, Karen Onel, Suzanne C. Li, MD, Alexei A. Grom, Janalee Taylor, Hermine I. Brunner, Jennifer L. Huggins, James J. Nocton, Kathleen A. Haines, Barbara S. Edelheit, Michael Shishov, Lawrence K. Jung, Calvin B. Williams, Melissa S. Tesher, Denise M. Costanzo, Lawrence S. Zemel, Jason A. Dare, Murray H. Passo, Kaleo C. Ede, Judyann C. Olson, Elaine A. Cassidy, Thomas A. Griffin, Linda Wagner-Weiner, Jennifer E. Weiss, Larry B. Vogler, Kelly A. Rouster-Stevens, Timothy Beukelman, Randy Q. Cron, Daniel Kietz, Kara M. Schmidt, Kenneth Schikler, Jay Mehta, Tracy V. Ting, James W. Verbsky, Anne B. Eberhard, Bin Huang, Edward H. Giannini

United Kingdom Juvenile Idiopathic Arthritis Genetics Consortium (UKJIAGC): Eileen Baildam, Annette Bryant, Lindsay Cameron, Joyce Davidson, Rachelle Donn, Helen Foster, Paul Gilbert, Vikki Gould, Helen Hanson, Wheldon Houlsby, Emma Inness, Olivia Lloyd, Carol Lydon, Natasha Makengo, Alexandra Meijer, Jill Paxton, Liang Qiao, Kay Riding, Tauny Southwood, Wendy Thomson, Debbie Wade, Peter Ward, Lucy R Wedderburn, Nick Wilkinson, Patricia Woo.

Cohort information

The post QC US cohorts comprised 2546 US JIA patients and 4386 US controls. Less than one half of these cases have already been included in a genome-wide association study and previously described[1,2]. Notably, 95 of these patients were from multiplex pedigrees such that for each

pedigree one RF negative polyarthritis or oligoarticular JIA case was randomly selected for genotyping. Clinics enrolling at least 5 JIA patients for Cincinnati-based studies (listed in order of number contributed) were located in Cincinnati, OH; Atlanta, GA; Columbus, OH; Little Rock, AR; Long Island, NY; Charlotte, NC; Cleveland, OH; Chicago, IL; Dover, DE; Nashville, TN; Philadelphia, PA; Milwaukee, WI; Toledo, OH; Charleston, SC; and Indianapolis, IN. Additional DNA from JIA cases collected independently by investigators in Salt Lake City, UT (367 cases, where about 75% overlap with replication cohort in previous GWAS studies[1,2]), Dallas, TX (86 cases), Kansas City, MO (74 cases) and Boston, MA (13 cases) or enrolled as part of the Trial of Early Aggressive Therapy in Juvenile Idiopathic Arthritis (TREAT) study (clinical trials identifier NCT00443430) (22 cases) were made available for genotyping in Cincinnati.

The US controls were derived from four sources: 793 healthy children without known major health conditions recruited from the geographical area served by Cincinnati Children's Hospital Medical Center (CCHMC) and 119 healthy adults collected at CCHMC. Previous JIA GWAS studies have include about 75% of only the pediatric controls, 484 healthy adult controls from Utah screened for autoimmune diseases and all were included in the replication cohort of previous GWAS studies[1,2]. 848 healthy adult controls collected at the Oklahoma Medical Research Foundation; and 1804 healthy US adult controls from the Genotype and Phenotype registry (www.gapregistry.org) and the NIDDK IBD Genetics Consortium. Healthy controls from the Oklahoma Medical Research Foundation (OMRF) were provided by the Lupus Family Registry and Repository (LFRR)[3] and the Oklahoma Immune Cohort (OIC). Each individual completed the Connective Tissue Disease Screening Questionnaire (CSQ)[4] and individuals with a "probable" systemic rheumatic disease were excluded. Each individual was enrolled into these studies after appropriate written consent and IRB approval by the OMRF and the University of Oklahoma Health Sciences Center. Healthy controls were also provided from the University of Minnesota SLE sibship collection[5] and these subjects were enrolled after appropriate written consent and IRB approval by the University of Minnesota.

The US collections and their use in genetic studies have been approved by the Institutional Review Board of CCHMC and each collaborating center.

The post QC UK cohort comprised 1636 JIA patients from five sources: The British Society for Paediatric and Adolescent Rheumatology (BSPAR) National Repository of JIA; a cohort of UK patients with long-standing JIA, described previously[6]; a cohort collected as part of the Childhood Arthritis Prospective Study (CAPS), a prospective inception cohort study of JIA cases from 5 centers across UK[7]; a cohort of children recruited for the SPARKS-CHARM (Childhood Arthritis Response to Medication) study, who fulfil ILAR criteria for JIA and are about to start new disease-modifying

medication for active arthritis[8] and an ongoing collection of UK cases, the UK JIA Genetics Consortium (UKJIAGC). JIA cases were classified according to ILAR criteria[9]. Of these, 383 have been newly genotyped compared to the original Immunochip paper[10]. There is overlap in the JIA cases used in this study and in previous UK candidate gene studies of JIA[11-14]. All UK JIA cases were recruited with ethical approval and provided informed consent [North-West Multi-Centre Research Ethics Committee (MREC 99/8/84), the University of Manchester Committee on the Ethics of Research on Human Beings and National Research Ethics Service (NRES 02/8/104)]. The 8579 UK controls comprised the shared UK 1958 Birth cohort and UK Blood Services Common Controls. The collection was established as part of the WTCCC[15].

The post QC German cohort comprised 450 German JIA patients and 480 controls. These cases have already been included as a replication cohort in a genome-wide association study and previously described. These patients were recruited from the German Center for Rheumatology in Children and Adolescents, Garmisch-Partenkirchen; the Department of Pediatrics, University of Tübingen; Children's Rheumatology Unit Sendenhorst, Germany; and the Department of Pediatrics, University of Prague, Czech Republic. JIA was determined retrospectively by chart review. German population-based control samples were prepared from cord blood obtained from healthy newborns in the Survey of Neonates in Pomerania (SNiP) consortium[16]. The respective Institutional Review Boards approved the collection of these samples and participation in this study.

The post QC Norwegian cohort comprised 271 Norwegian JIA patients and 945 controls. The patient cohort comprised consecutive cases of JIA from two defined geographical areas in Norway with disease onset from 2000-2012 and with available DNA samples. The study was an extended part of the Nordic JIA Study, a prospective, multicentre cohort study initiated by NoSPeR (Nordic Study group of Pediatric Rheumatology) as previously described[17]. The cohort aimed to be as close to population-based as possible, as centres participated only if they were able to include all children diagnosed with JIA in their catchment area. Approval from medical and health research ethical committees and data authorities were granted and informed consent obtained from parents and/or children according to national regulations. The Norwegian controls were from the second (1995-1997) or third (2006-2008) waves of the population-based Nord-Trøndelag Health Study (HUNT) (www.ntnu.edu/hunt). They were a random selection of participants who had answered "no" to questions of whether they had ever been given a diagnosis of rheumatoid arthritis or ankylosing spondylitis. All HUNT participants gave written, informed consent. The study was approved by the Regional Committee for Medical and Health Research Ethics, Central Norway, the Norwegian Data Inspectorate, and the Norwegian Department of Health.

The post QC Canadian Cohort comprised 140 Canadian JIA patients. The BBOP study (Biologically Based Outcome Predictors in JIA) is a multi-center inception cohort of prospectively collected children with new onset JIA recruited from 11 Canadian Pediatric Rheumatology Programs. Children were included in the study if they satisfied the ILAR classification criteria, were within six months of disease onset, and were treatment-naïve for medications except for non-steroidal anti-inflammatory agents. Peripheral blood and saliva samples were collected and processed as per standardized protocol at enrolment and transported to the central biobank (Sickkids, Toronto, ON). Informed consent for participation was obtained from parents and informed consent or assent was obtained from patients as applicable. The study was approved by the Research Ethics Board at each participating site.

Reference List

1. Thompson,S.D. *et al.* The susceptibility loci juvenile idiopathic arthritis shares with other autoimmune diseases extend to PTPN2, COG6, and ANGPT1. *Arthritis Rheum.* **62**, 3265-3276 (2010).

2. Thompson,S.D. *et al.* Genome-wide association analysis of juvenile idiopathic arthritis identifies a new susceptibility locus at chromosomal region 3q13. *Arthritis Rheum.*(2012).

3. Rasmussen,A. *et al.* The lupus family registry and repository. *Rheumatology. (Oxford)* **50**, 47-59 (2011).

4. Karlson,E.W. *et al.* A connective tissue disease screening questionnaire for population studies. *Ann. Epidemiol.* **5**, 297-302 (1995).

5. Gaffney,P.M. *et al.* Genome screening in human systemic lupus erythematosus: Results from a second Minnesota Cohort and combined analyses of 187 sib-pair families. *American journal of Human Genetics* **66**, 547-556 (2000).

6. Packham,J.C. & Hall,M.A. Long-term follow-up of 246 adults with juvenile idiopathic arthritis: functional outcome. *Rheumatology. (Oxford)* **41**, 1428-1435 (2002).

7. Adib,N. *et al.* Association between duration of symptoms and severity of disease at first presentation to paediatric rheumatology: results from the Childhood Arthritis Prospective Study. *Rheumatology. (Oxford)* **47**, 991-995 (2008).

8. Moncrieffe,H. *et al.* Generation of novel pharmacogenomic candidates in response to methotrexate in juvenile idiopathic arthritis: correlation between gene expression and genotype. *Pharmacogenet. Genomics* **20**, 665-676 (2010).

9. Petty,R.E. *et al.* International League of Associations for Rheumatology classification of juvenile idiopathic arthritis: second revision, Edmonton, 2001. *J. Rheumatol.* **31**, 390-392 (2004).

10. Hinks,A. *et al.* Dense genotyping of immune-related disease regions identifies 14 new susceptibility loci for juvenile idiopathic arthritis. *Nat. Genet.* **45**, 664-669 (2013).

11. Hinks,A. *et al.* Association of the IL2RA/CD25 gene with juvenile idiopathic arthritis. *Arthritis Rheum.* **60**, 251-257 (2009).

12. Hinks,A. *et al.* Association of the CCR5 gene with juvenile idiopathic arthritis. *Genes Immun.* **11**, 584-589 (2010).

13. Hinks,A. *et al.* Association of the AFF3 gene and IL2/IL21 gene region with juvenile idiopathic arthritis. *Genes Immun.* **11**, 194-198 (2010).

14. Hinks,A. *et al.* Overlap of disease susceptibility loci for rheumatoid arthritis and juvenile idiopathic arthritis. *Ann. Rheum. Dis.* **69**, 1049-1053 (2010).

15. The Wellcome Trust Case Control consortium Genome-wide association study of 14,000 cases of seven common diseases and 3,000 shared controls. *Nature* **447**, 661-678 (2007).

16. Beyersdorff,A. *et al.* Survey of Neonates in Pomerania (SniP): a population based analysis of the mothers' quality of life after delivery with special relations to their social integration. *Int. J. Public Health* **53**, 87-95 (2008).

17. Nordal,E. *et al.* Ongoing disease activity and changing categories in a long-term nordic cohort study of juvenile idiopathic arthritis. *Arthritis Rheum.* **63**, 2809-2818 (2011).

Supplementary Tables

Population sample	Start sample size	<98% CF and autosomal heterozygosity	Fail IBD	Fail Admixture	Additional exclusions*	Final sample size
US cases	2863	22	159	26	110	2546
US controls	5985	421	79	1068	31	4386
UK cases	1904	62	121	27	58	1636
UK controls	8940	110	241	10	0	8579
German cases	519	28	1	0	40	450
German controls	489	7	1	1	0	480
Norwegian cases	285	10	2	1	1	271
Norwegian controls	989	10	10	3	21	945
Canadian cases	166	1	2	21	2	140
**Total cases**	**5737**	**123**	**285**	**75**	**203**	**5043**
**Total controls**	**16403**	**548**	**331**	**1082**	**30**	**14390**

Supplementary Table 1 Sample quality control by population sample for data missingness (call frequency), autosomal heterozygosity, admixture testing and identity-by-descent (IBD) with numbers pre and post QC with breakdown by QC stage.

*additional exclusions due to gender mismatches or misdiagnosis of cases.

CF=call frequency

Population sample	Numbers	Gender		ILAR Category							
		Female (%)	Male (%)	Systemic JIA (%)	Persistent oligoarthritis (%)	Extended Oligoarthritis (%)	RF negative polyarthritis (%)	RF positive polyarthritis (%)	Enthesitis-related arthritis (%)	Juvenile psoriatic arthritis (%)	Undefined JIA (%)
US cases	2546	1930 (75.9)	614 (24.1)	156 (6.1)	930 (36.5)	285 (11.2)	891 (35)	219 (8.6)	56 (2.2)	5 (0.2)	4 (0.2)
US controls	4386	2638 (60.1)	1748 (39.9)								
UK cases	1636	1098 (67.1)	538 (32.9)	196 (12.0)	442 (27)	281 (17.2)	397 (24.3)	94 (5.7)	80 (4.9)	89 (5.4)	57 (3.5)
UK controls	8579	4535 (52.9)	4044 (47.1)								
German cases	450	333 (74)	117 (26)	-	275 (61.1)	55 (12.2)	119 (26.4)	1 (0.2)	-	-	-
German controls	480	232 (48.3)	248 (51.7)								
Norwegian Cases	271	174 (64.2)	97 (35.8)	4 (1.5)	82 (30.3)	37 (13.7)	57 (21)	13 (4.8)	34 (12.5)	8 (3)	36 (13.3)
Norwegian controls	945	589 (62.3)	356 (37.7)								
Canadian cases	140	92 (65.7)	48 (34.3)	17 (12.1)	22 (15.7)	-	61 (43.6)	10 (7.1)	13 (9.3)	10 (7.1)	7 (5)
**Total cases**	**5043**	**3627 (72)**	**1414 (28)**	**373 (7.4)**	**1751 (34.7)**	**658 (13)**	**1525 (30.2)**	**337 (6.7)**	**183 (3.6)**	**112 (2.2)**	**104 (2.1)**
**Total controls**	**14390**	**7994 (55.6)**	**6396 (44.4)**								

Supplementary Table 2 Sample Collections with numbers post QC with breakdown by gender and ILAR category.

Trait 1	Number cases	Number controls	Trait 2	Number cases	Number controls	h^2_{SNP} (SE) trait 1 All Immunochip	h^2_{SNP} (SE) trait 2 All Immunochip	rG	h^2_{SNP} (SE) trait 1 HLA region	h^2_{SNP} (SE) trait 2 HLA region	rG
POligo	1751	7195	EOligo	658	7195	0.19 (0.01)	0.31 (0.02)	**0.96 (0.02)**	0.09 (0.009)	0.16 (0.02)	**0.98 (0.01)**
POligo	1751	7195	RF-poly	1525	7195	0.19 (0.01)	0.16 (0.01)	**0.88 (0.03)**	0.09 (0.01)	0.06 (0.008)	**0.87 (0.04)**
POligo	1751	7195	RF+poly	337	7195	0.19 (0.01)	0.28 (0.03)	0.33 (0.06)	0.08 (0.01)	0.08 (0.01)	0.13 (0.12)
POligo	1751	7195	SysJIA	373	7195	0.19 (0.01)	0.06 (0.02)	0.42 (0.12)	0.08 (0.009)	0.02 (0.007)	0.44 (0.16)
POligo	1751	7195	ERA	183	7195	0.19 (0.01)	0.87 (0.07)	0.27 (0.05)	0.08 (0.02)	0.52 (0.06)	0.27 (0.09)
POligo	1751	7195	jPsA	112	7195	0.19 (0.01)	0.19 (0.07)	0.58 (0.13)	0.09 (0.01)	0.05 (0.02)	**0.81 (0.17)**
EOligo	658	7195	RF-poly	1525	7195	0.35 (0.02)	0.16 (0.01)	**0.89 (0.03)**	0.16 (0.02)	0.06 (0.008)	**0.94 (0.03)**
EOligo	658	7195	RF+poly	337	7195	0.37 (0.03)	0.29 (0.03)	0.39 (0.06)	0.16 (0.02)	0.08 (0.02)	0.15 (0.13)
EOligo	658	7195	SysJIA	373	7195	0.36 (0.02)	0.07 (0.02)	0.52 (0.12)	0.16 (0.02)	0.02 (0.008)	0.31 (0.18)
EOligo	658	7195	ERA	183	7195	0.36 (0.02)	0.88 (0.07)	0.27 (0.05)	0.16 (0.02)	0.53 (0.06)	0.21 (0.1)
EOligo	658	7195	jPsA	112	7195	0.36 (0.02)	0.21 (0.06)	0.76 (0.13)	0.17 (0.02)	0.06 (0.02)	**0.88 (0.11)**
RF-poly	1525	7195	RF+poly	337	7195	0.19 (0.01)	0.29 (0.03)	0.35 (0.06)	0.06 (0.009)	0.08 (0.02)	0.17 (0.13)
RF-poly	1525	7195	SysJIA	373	7195	0.18 (0.01)	0.06 (0.02)	0.61 (0.13)	0.06 (0.009)	0.02 (0.007)	0.64 (0.15)
RF-poly	1525	7195	ERA	183	7195	0.18 (0.01)	0.88 (0.06)	0.22 (0.05)	0.06 (0.009)	0.51 (0.06)	0.16 (0.1)
RF-poly	1525	7195	jPsA	112	7195	0.18 (0.01)	0.21 (0.07)	0.72 (0.13)	0.06 (0.009	0.05 (0.02)	**0.93 (0.1)**
RF+poly	337	7195	SysJIA	373	7195	0.27 (0.03)	0.07 (0.02)	0.45 (0.14)	0.07 (0.01)	0.02 (0.008)	0.19 (0.2)
RF+poly	337	7195	ERA	183	7195	0.27 (0.03)	0.89 (0.07)	0.12 (0.07)	0.07 (0.01)	0.54 (0.07)	0.07 (0.13)
RF+poly	337	7195	jPsA	112	7195	0.27 (0.03)	0.21 (0.07)	0.22 (0.14)	0.07 (0.01)	0.05 (0.02)	0.14 (0.23)
SysJIA	373	7195	ERA	183	7195	0.07 (0.02)	0.88 (0.07)	0.13 (0.12)	0.02 (0.008)	0.53 (0.07)	0.1 (0.18)
SysJIA	373	7195	jPsA	112	7195	0.07 (0.02)	0.22 (0.07)	0.56 (0.23)	0.02 (0.008)	0.05 (0.02)	0.45 (0.27)
ERA	183	7195	jPsA	112	7195	0.89 (0.07)	0.22 (0.07)	0.44 (0.13)	0.59 (0.07)	0.05 (0.02)	0.54 (0.17)

h^2_{SNP}= Heritability, SE=Standard error, rG=genetic correlation

Supplementary Table 3 Results of the bivariate analysis using GCTA comparing genetic correlation between JIA categories.

Cohort	h^2_{SNP}(SE) All Immunochip	h^2_{SNP}(SE) HLA region
All JIA	0.13 (0.005)	0.06 (0.007)
Persistent oligoarthritis	0.22 (0.01)	0.13 (0.01)
Extended oligoarthritis	0.4 (0.02)	0.22 (0.03)
RF negative polyarthritis	0.2 (0.01)	0.09 (0.01)
RF positive polyarthritis	0.24 (0.03)	0.08 (0.02)
Systemic JIA	0.05 (0.02)	0.02 (0.008)
ERA	0.94 (0.07)	0.68 (0.09)
Juvenile PsA	0.22 (0.07)	0.05 (0.02)
Combined oligoarthritis and RF negative polyarthritis dataset	0.17 (0.006)	0.08 (0.007)

h^2_{SNP}= Heritability, SE=Standard error

Supplementary Table 4 Heritability estimates for HLA and All Immunochip in all JIA and its categories

	Persistent oligoarthritis				Extended oligoarthritis				RF negative polyarthritis				RF positive polyarthritis			
**Amino acid residue**	**MAF cases**	**MAF controls**	**p-value**	**OR 95% CI**	**MAF cases**	**MAF controls**	**p-value**	**OR 95% CI**	**MAF cases**	**MAF controls**	**p-value**	**OR 95% CI**	**MAF cases**	**MAF controls**	**p-value**	**OR 95% CI**
HLA-DRB1 AA pos 13 Serine	0.46	0.34	ref		0.44	0.34	ref		0.42	0.34	ref		0.27	0.34	ref	
HLA-DRB1 AA pos 13 arginine	0.10	0.15	1.61×10^{-25}	0.52 0.45-0.58	0.09	0.15	1.71×10^{-11}	0.50 0.41-0.61	0.11	0.15	3.24×10^{-14}	0.61 0.54-0.69	0.09	0.15	7.77×10^{-02}	0.77 0.57-1.02
HLA-DRB1 AA pos 13 histidine	0.07	0.19	1.80×10^{-63}	0.30 0.27-0.35	0.07	0.19	9.27×10^{-26}	0.30 0.24-0.37	0.11	0.19	9.52×10^{-29}	0.49 0.43-0.55	0.36	0.19	1.54×10^{-18}	2.44 2.00-2.97
HLA-DRB1 AA pos 13 Tyrosine	0.05	0.14	9.53×10^{-56}	0.27 0.23-0.32	0.06	0.14	8.35×10^{-20}	0.32 0.25-0.41	0.08	0.14	9.63×10^{-27}	0.46 0.39-0.53	0.06	0.14	2.23×10^{-04}	0.52 0.36-0.73
HLA-DRB1 AA pos 13 Phenylalanine	0.15	0.14	3.98×10^{-04}	0.82 0.73-0.91	0.17	0.14	6.71×10^{-01}	0.97 0.82-1.13	0.16	0.14	6.10×10^{-01}	0.97 0.87-1.09	0.16	0.14	1.92×10^{-03}	1.47 1.15-1.88
HLA-DRB1 AA pos 13 glycine	0.17	0.05	3.37×10^{-59}	2.73 2.41-3.08	0.17	0.05	9.23×10^{-32}	2.87 2.40-3.42	0.12	0.05	6.53×10^{-24}	2.02 1.76-2.32	0.07	0.05	6.88×10^{-03}	1.63 1.13-2.29

Supplementary Table 5 Results by residue for DRB1 amino acid position 13 by ILAR category

Variant	position	p-value	Conditioned on
HLA-DRB1 AA pos 13	32660109	1.92×10^{-377}	Primary analysis
HLA-DRB1 AA pos 67	32659947	7.01×10^{-83}	HLA-DRB1 AA pos 13
HLA-DRB1 AA pos 181	32657335	3.33×10^{-22}	HLA-DRB1 AA pos 13 & 67
HLA-DRB1 AA pos 71	32659935	1.16×10^{-8}	HLA-DRB1 AA pos 13 & 67 & 181
HLA-DPB1*02:01	33157346	7.70×10^{-57}	2digit & 4digit DRB1 alleles
HLA-A AA pos 95	30019036	1.40×10^{-37}	2digit & 4digit DRB1 alleles & HLA-DPB1*02:01
HLA-B AA pos 152	31432015	5.31×10^{-10}	2digit & 4digit DRB1 alleles & HLA-DPB1*02:01 & HLA-A pos 95

Supplementary Table 6 Evidence for independent effects in the combined oligoarthritis and RF negative polyarthritis dataset

JIA category	HLA classical allele	HLA amino acid	Adult counterpart	HLA classical allele	HLA amino acid
Combined oligoarthritis and RF negative polyarthritis		HLA-DRB1 amino acid pos13 Glycine & Serine residue	Seronegative RA		HLA-DRB1 amino acid pos11 Serine & Leucine residue
RF positive polyarthritis		HLA-DRB1 amino acid pos13 Histidine residue	Seropositive RA		HLA-DRB1 amino acid pos11 Histidine residue
sJIA	HLA-DRB1*11		Stills disease	No data	No data
ERA	HLA-B*27		Ankylosing spondylitis	HLA-B*27	
jPsA	HLA-DQA1*0401		PsA	HLA-C*0602	

Supplementary Table 7 Comparison of HLA associations between JIA categories and adult rheumatic diseases

Supplementary Figure 1

Schematic flow diagram of the analysis procedure in this study

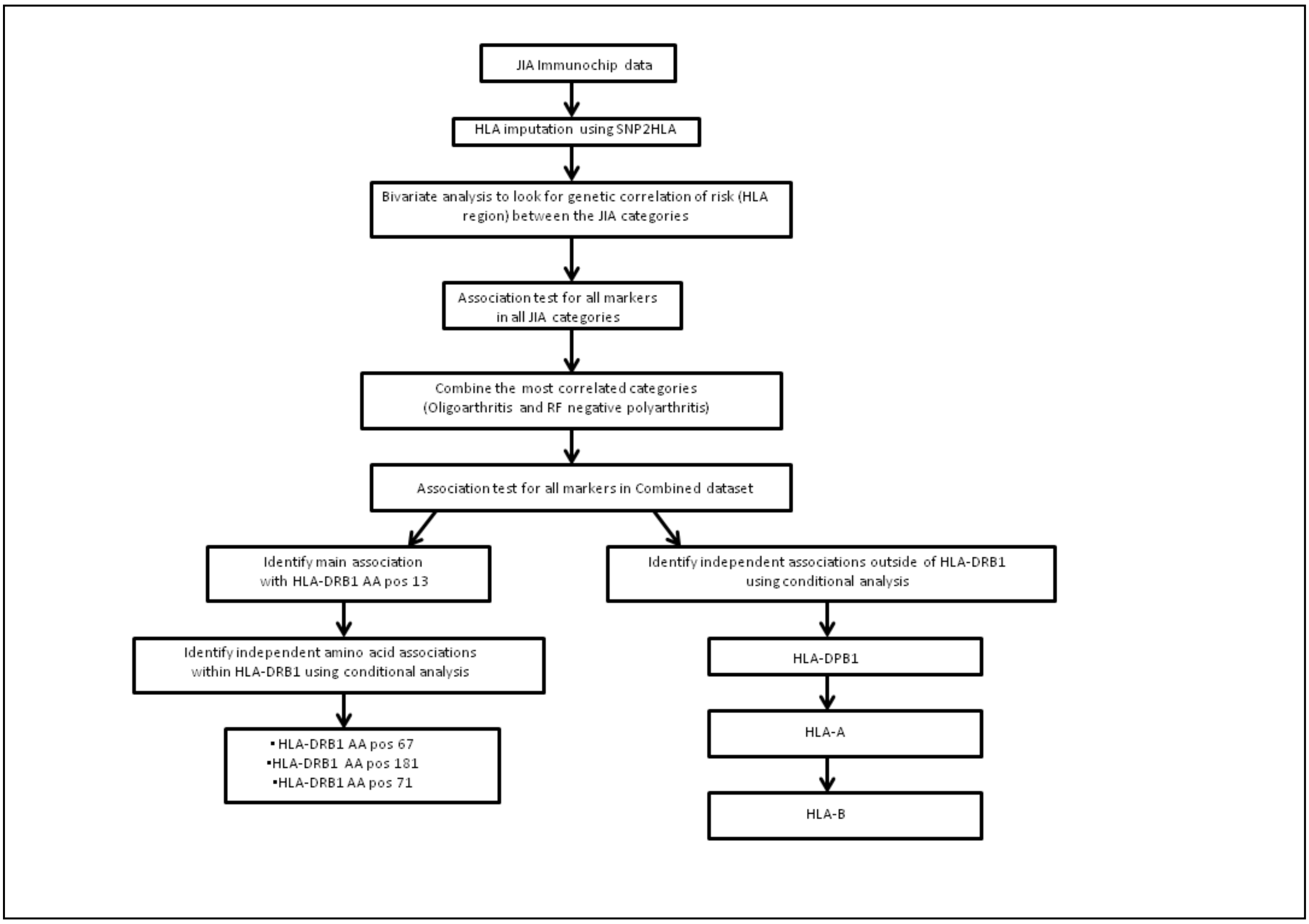

Supplementary Figure 2 Effect of individual amino acid residues within HLA-DRB1 amino acid position 13 for the associated JIA ILAR categories, the allele frequencies of cases (blue) and controls (red), the univariate odds ratios shown at the top of each bar. MAF= Minor allele frequency. Amino acid codes F=Phenylalanine, G=Glycine, H=Histidine, R=Arginine, S=Serine, Y=Tyrosine.

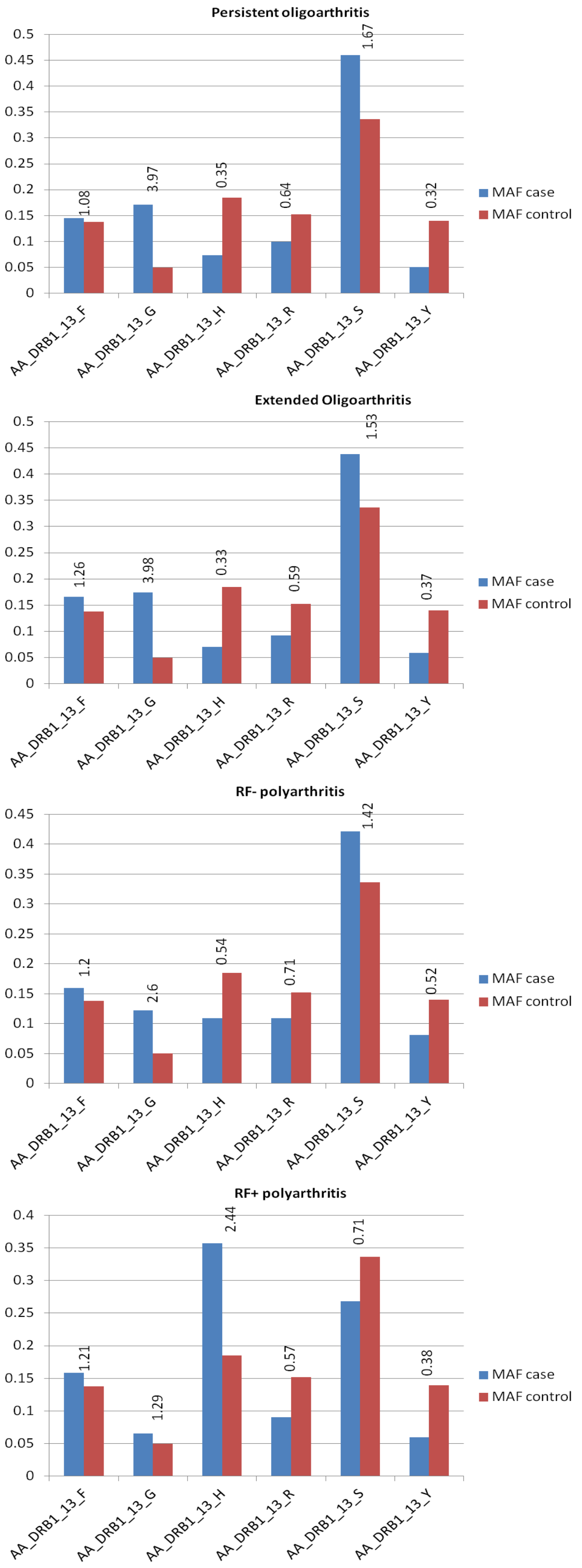

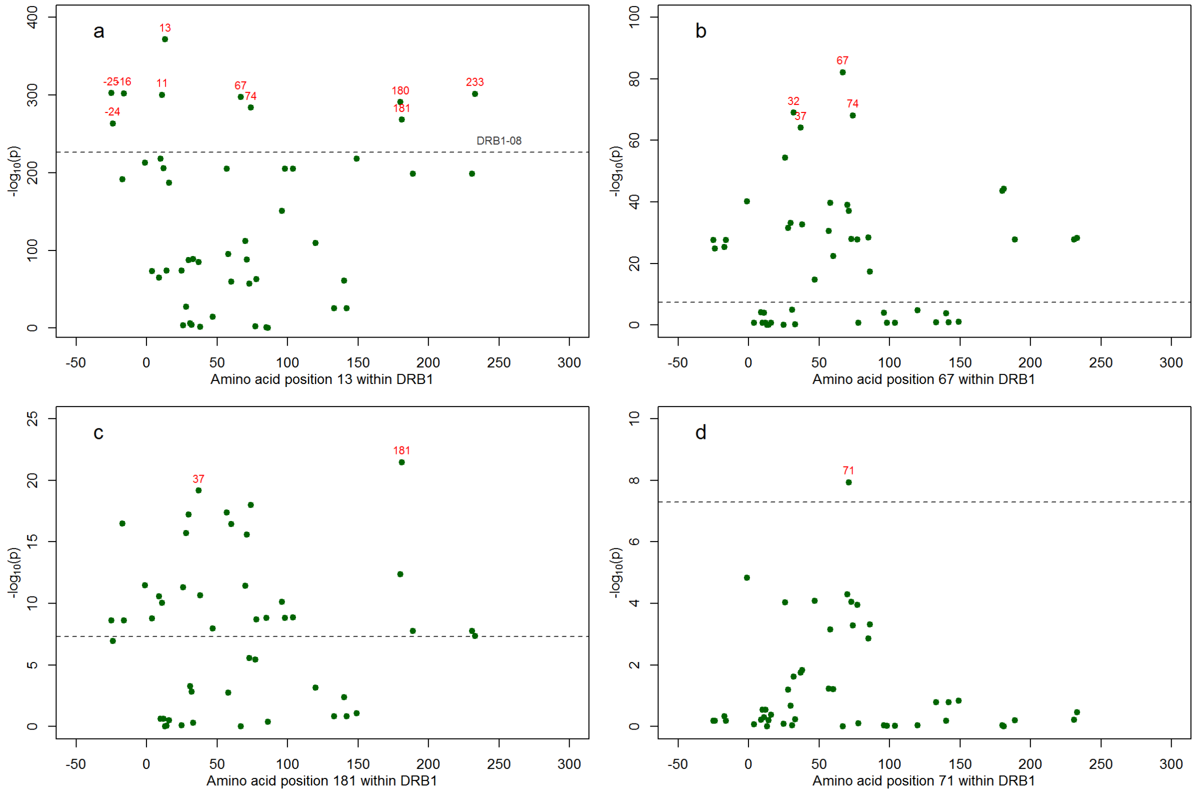

Supplementary Figure 3 Association results for amino acids in HLA-DRB1 in the combined oligoarthritis and RF negative polyarthritis dataset. (a) Amino acid position 13 showed the strongest association ($p < 10^{-377}$), the dashed vertical line shows the p-value for association of the classical HLA-DRB1*08 allele. Multiple amino acids show more significant association than this classical allele. (b) conditioning on position 13, position 67 was associated ($p < 10^{-83}$) the dashed vertical line shows the p-value for the genome-wide significance threshold ($P<5 \times 10^{-8}$). (c) conditioning on position 13 and 67, position 181 was associated ($p < 10^{-22}$) the dashed vertical line shows the p-value for the genome-wide significance threshold ($P<5 \times 10^{-8}$). (d) conditioning on position 13, 67 and 181, position 71 was associated ($p < 10^{-8}$) the dashed vertical line shows the p-value for the genome-wide significance threshold ($P<5 \times 10^{-8}$).

Supplementary Figure 4 Different effect sizes for amino acid residues at HLA-DRB1 position 13
Comparing Seropositive RA with the combined oligoarthritis and RF negative polyarthritis dataset (a) and RF positive polyarthritis (b).

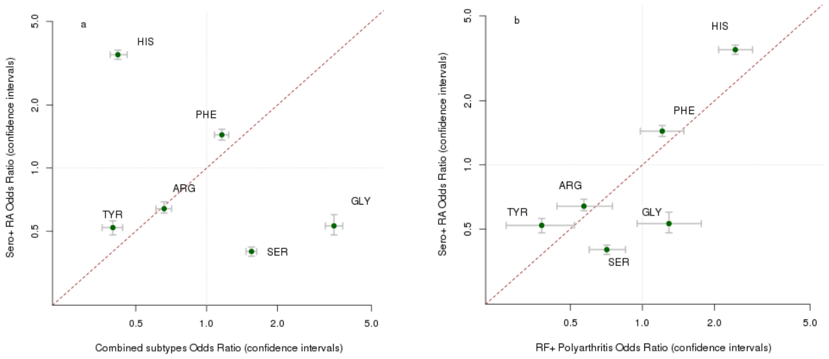

Comparing Seronegative RA with the combined oligoarthritis and RF negative polyarthritis dataset **(c)** and RF positive polyarthritis **(d)**.

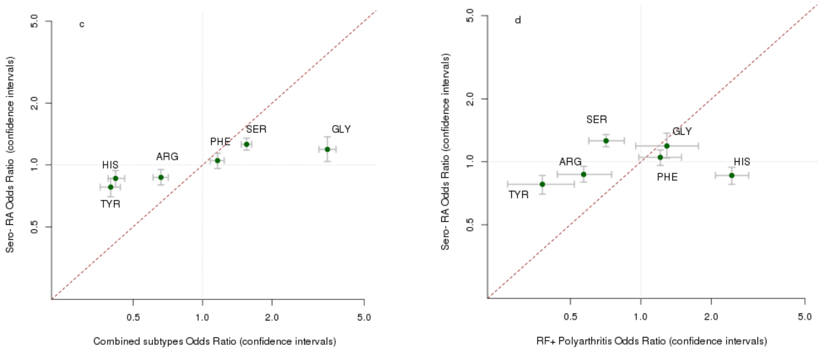

Supplement: supplementary data [file annrheumdis-2016-210025supp001.pdf]
